# Bone Perspectives in Functional Hypothalamic Amenorrhoea: An Update and Future Avenues

**DOI:** 10.3389/fendo.2022.923791

**Published:** 2022-06-20

**Authors:** Preeshila Behary, Alexander N. Comninos

**Affiliations:** ^1^Endocrine Bone Unit, Imperial College Healthcare NHS Trust, London, United Kingdom; ^2^Section of Endocrinology and Investigative Medicine, Imperial College London, London, United Kingdom; ^3^Department of Endocrinology, Imperial College Healthcare NHS Trust, London, United Kingdom

**Keywords:** functional hypothalamic amenorrhoea, bone mineral density, osteoporosis, fractures, HRT, IGF1, kisspeptin

## Abstract

One of the most important and potentially long-lasting detrimental consequences of Functional Hypothalamic Amenorrhoea (FHA) is on skeletal homeostasis. Beyond oestrogen deficiency, FHA is associated with a cascade of additional neuro-endocrine and metabolic alterations, some adaptive, but which combine to disrupt skeletal homeostasis. Ultimately, this leads to a two-fold increased risk of fractures in women with FHA compared to healthy eumenorrhoeic women. Although the cornerstone of management of FHA-related bone loss remains recovery of menses *via* restoration of metabolic/psychological balance, there is rapidly developing evidence for hormonal manipulations (with a particular emphasis on route of administration) and other pharmacological treatments that can protect or improve skeletal homeostasis in FHA. In this mini-review, we provide an update on the pathophysiology, clinical management and future avenues in the field from a bone perspective.

## Introduction

Functional Hypothalamic Amenorrhoea (FHA) results from the suppression of the hypothalamic control of the reproductive axis, resulting in the cessation of menses (in the absence of an organic cause). Negative energy conditions with weight loss, such as in Anorexia Nervosa (AN) or without significant weight loss (but low body fat) such as in training athletes, and psychological stress, are the main aetiologies predisposing to FHA. AN affects approximately 0.2-4% of women, with the majority experiencing amenorrhoea (1, 2). Indeed, in female athletes, the reported prevalence of secondary amenorrhoea is up to 60% (3).

Irrespective of the aetiology, FHA has detrimental effects on the skeleton through disruption of normal skeletal homeostasis, ultimately resulting in an increased risk of fractures. Therefore, it is crucial to fully appreciate the factors implicated in bone impairments in this condition. Importantly, different aetiologies of FHA and their time of onset (e.g. adolescent versus adult) are associated with characteristic neuroendocrine changes which have distinct effects on skeletal homeostasis and fracture risk (4, 5). Therefore, bone management may be tailored accordingly.

The aim of this mini-review is to give an overview of the effects of FHA on bone, specifically how they differ according to the underlying aetiology. Furthermore, we discuss current and future treatment avenues and identify gaps in the literature to inform future research, thereby providing an update for the field.

## Fha and Bone, According to Aetiology

**Figure 1:**
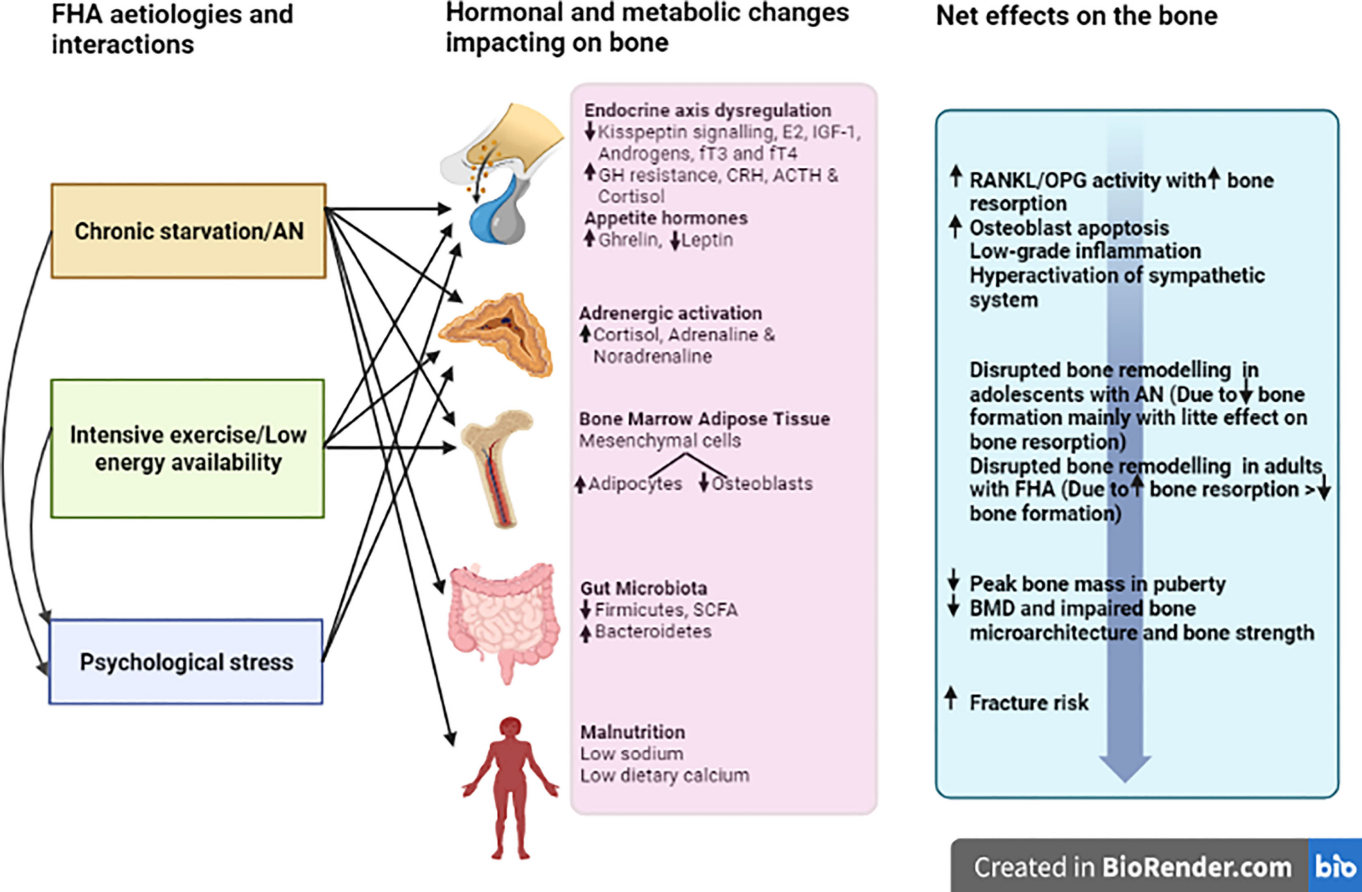
Pathophysiology and the effects of FHA on bone.

A summary of the aetiologies for FHA as discussed above, and their detrimental effects on the skeleton are displayed in **Figure 1**.

### Anorexia Nervosa

There is an abundance of evidence for low bone mineral density (BMD) and increased risk of fractures in FHA due to AN. In a cross-sectional study of 214 women with AN aged 17-45 years, over half had osteopenia and a third were osteoporotic. Furthermore, thirty percent of the cohort reported a previous fracture (6). This was replicated in a cohort of 60 adolescent girls with AN, where 52% had a reduced BMD (based on Z score of < -1) (7). Additionally, indices of bone quality at the microarchitectural level and bone strength, as assessed by High-Resolution peripheral Quantitative CT (HR-pQCT) were also negatively affected (8). In keeping with this high fracture risk, another study observed that the incidence rate of fracture in patients with AN (mean age 21.2 ± 9.2 years, with 94% of the cohort being female), is nearly doubled, compared to controls matched for age and gender. Furthermore, this increased risk persisted beyond 10 years from diagnosis suggesting irreversible bone impairment (9).

The issue of bone fragility is compounded by the fact that AN is predominantly a condition of younger women, typically in adolescence. This corresponds to a critical time for attaining peak bone mass (PBM). Indeed, most of the PBM is acquired before the age of 19 in females (10). In a long-term retrospective follow-up study of women who acquired AN during puberty (and therefore likely failed to achieve PBM), an increased risk of fractures was observed as far as 38 years after diagnosis, with a cumulative incidence risk of 57% at 40 years (11). In another study of over 400 participants, the lifetime prevalence of fractures was 59.8% higher in adolescents with AN, compared to healthy controls. Interestingly, this was not associated with any major reduction in axial BMD (12). A possible explanation relates to the limitation of using Dual-Energy X-ray Absorptiometry (DEXA) in this young cohort, where changes in bone microarchitecture and strength, are not adequately captured. These observations have important implications with regards to treatment strategies and the importance of early interventions to minimise long-term fracture risk.

Following on from this, it is relevant from a management perspective to note a key difference between adolescents and adults with AN, with respect to underlying bone turnover. Adolescent girls with AN, have reduced bone formation with fairly normal bone resorption, whereas adult women have reduced bone formation with markedly increased bone resorption. Overall this results in low remodelling in adolescents but higher remodelling in adult women with FHA (13). This difference suggests a limited benefit for the use of anti-resorptive agents in adolescence which has been borne out in clinical studies and highlights the different responses to treatment dependent on time of onset of FHA (14).

### Exercise

Exercise can be seen as a double-edged sword. In healthy populations, weight-bearing exercise has been shown to benefit BMD and a positive legacy effect is present in ex-athletes (15, 16). This was demonstrated in a study of 48 overweight adults randomised to either calorie restriction or weight-bearing exercise for a year. Despite comparable weight loss of around 8-10%, giving an approximate mean BMI of 24 kg/m^2^, only the calorie-restricted group experienced a reduction in lumbar BMD (mean 2.2%), suggesting a protective effect of exercise on BMD in the face of negative energy balance (17). However, it is clear that extensive exercise coupled with low energy intake can lead to FHA and bone loss, in the so-called Female Athlete Triad. This triad consists of 3 inter-linked conditions: low energy availability, menstrual disruption and low BMD (18). Studies suggest a minimum calorie intake threshold of approximately 30 kcal/kg lean body mass/day, is required to maintain reproductive axis function (19, 20). However, this concept has been disputed by others. Lieberman et al. did not identify a specific energy threshold that induced menstrual disturbances in their cohort of women (randomised into low, moderate or high energy deficit interventional groups). However, using the threshold of 30 kcal/kg lean body mass/day in their cohort, they estimated the probability of inducing menstrual disturbances to be over 50% (21). Therefore in practice, there seems to be a spectrum of energy balance set points, at which menstrual disruption occurs at an individual level, likely related to genetic and other individual factors (22).

Regarding lifetime fracture risk, this was almost double in athletes with amenorrhoea (AA) compared to athletes with eumenorrhoea (AE) and four-fold higher compared to non-athletes (NA), in a retrospective study of 175 women. Stress fractures occurred in 32% versus 5.9% versus 0% in the AA, AE and NA cohorts respectively. Furthermore, bone microarchitecture was more negatively affected in AA, especially in those who sustained multiple stress fractures highlighting the detrimental combination of excess exercise with amenorrhoea (23).

There are some salient differences worth noting in the bone sequelae of FHA depending on an AN or exercise aetiology. In a recent study, Kandemir et al. compared bone parameters in women with AN (with or without amenorrhoea) to normal-weight athletes with oligomenorrhoea (AO) and normal-weight eumenorrhoeic controls. They observed a lower BMD and greater impairment of bone microarchitecture at all sites assessed in the AN group, compared to AO and control groups. The AO group demonstrated a lower BMD at the lumbar spine only relative to controls, and bone microarchitectural parameters were less impaired, especially at the weight-bearing tibia compared to the AN group. This highlights the greater severity of bone impairments in AN, relative to a protective bone effect during weight-bearing exercise with weight preservation, despite oligomenorrhoea. However, fracture rates were similar in AN and AO, although the latter displayed a predilection for stress fractures (which athletes are inherently more at risk of). Indeed, stress fractures were 15 times higher in the AO group compared to controls, and 7.5 times higher in the AO group compared to AN (4). Limitations of this study included its cross-sectional design and self-reporting of fractures. However, it undoubtedly highlights the different severities of bone impairment depending on underlying aetiology of amenorrhoea/oligomenorrhoea.

### Psychological Stress

Psychological stress is an under-appreciated but important cause of FHA. Psychological stress can independently suppress the reproductive axis but commonly co-exists and interacts synergistically with other stressors such as energy restriction and over-exercising (as above), resulting in FHA. In a recent study involving 61 exercising women by Strock et al, women with amenorrhoea showed a greater drive for thinness and a greater need for social approval than women with eumenorrhoea. Furthermore, this was positively associated with indicators of psychological stress and depression, assessed by questionnaires. This was despite both groups having comparable exercise intensity and energy intake, thus highlighting the role of stress in FHA (24). Others have also reported that women with FHA have more dysfunctional attitudes (such as drive for perfectionism, rigidity of ideas, preoccupation of being judged), more depressive symptoms and are less able to cope with stressors than eumenorrhoeic controls. These specific personality traits of women with FHA, therefore make them more susceptible to life stressors (25, 26).

These studies demonstrate an association of psychological stress with FHA but do not identify causality. However, psychological stress is a key activator of the Hypothalmic-Pituitary-Adrenal (HPA) axis, promoting cortisol secretion, which in excess has established negative effects on skeletal homeostasis (and reproductive function). However, there exist additional mechanisms linking psychological stress with bone disruption. Low grade inflammation as evidenced by increased pro-inflammatory markers (such as tumour necrosis alpha-α), has been associated with acute stress and shown to cause upregulation of Receptor activator nuclear factor kappa-B ligand (RANKL) signalling and therefore increased bone resorption in pre-clinical studies (27, 28). Stress-induced hyperactivation of the sympathetic system has also been proposed as another mechanism. Indeed, receptors for noradrenaline are present on osteoclasts and osteoblasts (29) and stress-induced bone loss is observed in the context of elevated noradrenaline levels in mice, while propranolol, a β-adrenergic antagonist, blocks this negative effect (30). Taken together, there is not only evidence that psychological stress can cause FHA as well as associate with AN/exercise, but that psychological stress itself can directly impair skeletal homeostasis.

## Endocrine Mediators of Bone Loss in Fha

The key defects in FHA are attenuated hypothalamic secretion of kisspeptin and downstream gonadotropin-releasing hormone (GnRH) (31). This results in inadequate secretion of downstream follicle-stimulating hormone and luteinising hormone to sustain normal menstrual cyclicity. The negative energy balance, low body fat and/or psychological stress result in the disruption of multiple neuro-endocrine signals (**Figure 1**) leading to failure of the downstream reproductive axis culminating in oestrogen deficiency and detrimental effects on skeletal homeostasis (32).

### Reduced Kisspeptin

Kisspeptin (secreted by kisspeptin neurons) is the master hypothalamic regulator of the reproductive axis and controls downstream GnRH secretion through kisspeptin receptors located upon GnRH neurons (33). In FHA, kisspeptin secretion has recently been shown to be reduced (34), while conversely administration of kisspeptin to patients with FHA can restore downstream pulsatile LH secretion (35). Crucially, kisspeptin neurons receive multiple neuro-endocrine and metabolic signals that can be disrupted in FHA, and so serve to orchestrate the downstream reproductive axis based on these inputs. Although kisspeptin secretion regulates downstream classical reproductive hormones crucial to skeletal homeostasis (predominantly oestrogen and testosterone), recent data has identified direct positive effects for kisspeptin in bone (36–39). However, although in FHA there is reduced kisspeptin signalling in the hypothalamus, it is currently unknown if kisspeptin signalling is also reduced in bone.

### Reduced Oestrogen

Oestrogen receptors are present on the three main bone cells: osteoclasts, osteoblasts and osteocytes. Oestrogen inhibits bone resorption directly by inducing osteoclastic apoptosis and indirectly by disrupting the RANKL/Osteoprotegerin (OPG) pathway. Recent work suggests that RANKL expression on bone lining cells (derived from osteoblasts) is a key mediator of oestrogen-controlled bone resorption (40). In addition, further new data has identified oestrogen-induced secretion of semaphorin 3A, a protein known to reduce bone resorption and increase bone formation, from osteocytes (41), as well as anti-apoptotic effects by oestrogen on osteoblasts (via promotion of autophagy) (42). Taken together, the net effect of oestrogen is a reduction in bone remodelling (due to greater effect on reducing bone resorption compared to increasing bone formation). Therefore, oestrogen deficiency states are characterised by increased bone remodelling resulting in disrupted skeletal homeostasis. In the early menopause transition, BMD decreases by about 2% per year (43). Further demonstrating the impact of oestrogen deficiency, eumenorrhoeic women with AN have higher BMD than amenorrhoeic (i.e. lower oestrogen levels) women with AN, although both groups display lower than normal BMD (T score -1.2 in eumenorrhoeic versus -2.3 in amenorrhoeic women) (44). This highlights the dominating detrimental impact of oestrogen deficiency as seen in FHA beyond other nutritional and endocrine effects of anorexia nervosa.

### Reduced Androgens

Low levels of testosterone and DHEA are observed in AN (45) with associated impairments in bone microarchitecture (46). However conflicting findings of high or normal levels of androgens have been observed in athletes and normal-weight women with FHA (45–47). Although androgens mediate most of their effect on bone indirectly from aromatisation into oestrogens, androgens themselves are also important in women predominantly for trabecular bone (48).

### Reduced Leptin

Leptin is reduced in FHA mainly secondary to acute calorie restriction and stress, independent of weight loss (49, 50). Leptin has both central and peripheral actions on bone. Centrally, low leptin levels reduce the secretion of Insulin Growth Factor-1 (IGF-1), oestrogen and thyroid hormones, which all normally have positive bone effects (51). These hormonal reductions are part of a necessary adaptive energy-sparing response, to minimise growth, reproduction and metabolism respectively. Peripherally, leptin receptors are present on osteoblasts with possible anabolic roles in bones by enhancing osteoblast proliferation (52, 53). Furthermore, *in vitro* studies suggest a role for leptin-driven differentiation of human marrow stem cells into osteoblasts further supporting an anabolic role (54).

### Elevated Ghrelin

In contrast to leptin, ghrelin levels are elevated in women with FHA (55). This response is presumed to be physiological to stimulate calorie intake and restore energy balance. Ghrelin is also a known growth hormone (GH) secretagogue and may contribute to excess GH secretion in AN (55). Interestingly, elevated ghrelin levels have been associated with a delayed return to menstrual cyclicity in women with persistent disordered eating in FHA, despite normalisation of weight and leptin levels. This suggests a direct effect of ghrelin on the reproductive axis in FHA (56). From a bone perspective, ghrelin directly stimulates osteoblast proliferation *in vitro*, and increases BMD in rodents *in vivo* (57). A similar anabolic effect on bone has also been observed following intracerebroventricular administration of ghrelin to rodents, independent of body weight (58). Taken together, these studies suggest central and peripheral positive effects of ghrelin on bone. However, although in FHA, ghrelin levels may be raised, this beneficial effect is far outweighed by the repercussions of other hormonal changes such as hypoestrogenism on bones.

### Elevated GH and Reduced IGF-1

IGF-1 levels are reduced by up to 50% in AN despite increased GH, in keeping with a state of GH resistance (59, 60). IGF-1 has established anabolic effects on bone through increases in osteoblast activity and collagen synthesis (61). Crucially, IGF-1 has a key role in the gain of bone mass during puberty and correlates positively with BMD and bone formation markers in adolescent girls with AN and with bone microarchitecture in adult women with AN (62, 63). This further highlights the potential longer-term detrimental effects of FHA on bone when there is failure to achieve an optimal PBM in younger years.

### Elevated Cortisol

Increased levels of Corticotrophin-Releasing Hormone (CRH), corticotrophin (ACTH) and downstream 24-hour cortisol levels are a consistent feature of FHA (32, 64). This is due to physical or psychological stress activating the HPA axis with the increases in cortisol capable of further suppressing the reproductive axis (32).

Hypercortisolaemia itself can contribute to bone loss. In a study of normal-weight and AN-induced adult women with FHA, hypercortisolaemia was observed in both groups, and was negatively correlated with BMD (65). There are multiple mechanisms for the detrimental effects of glucocorticoids (such as cortisol) on bone beyond the scope of this mini-review but include reduced gut absorption and increased renal loss of calcium, as well as increased osteoblast apoptosis and enhanced bone resorption *via* the RANKL/OPG pathway (66).

### Reduced Thyroid Hormones

AN is associated with reduced levels of free T3 (fT3) and free T4 (fT4) compared to controls, similar to the nonthyroidal illness syndrome observed in patients with systemic illness (67). Similarly, lower thyroidal hormonal levels have been reported in FHA due to exercise, compared to their eumenorrhoeic counterparts. In this study, reduced T3 and T4 levels were associated with a prolonged post-exercise muscle recovery rate, as assessed by phosphate recovery kinetics (68). In a more recent study involving women with FHA (but not AN), those with fT3 levels below the normal range had a lower BMD at the spine and hip as well as lower circulating osteocalcin levels (a marker of osteoblastic activity), compared to those with preserved fT3 levels (mean lumbar T score range: -0.6 to -3.4 versus 0.2 to -2.9 respectively; mean hip T score range: -0.4 to -2 versus 1.8 to -1.6 respectively). A compensatory increase in oxidative stress, driven by low fT3 levels, has been proposed as the underlying mechanism impairing skeletal homeostasis (69).

### Increased Bone Marrow Adipose Tissue

Bone marrow adipose tissue (BMAT) is increased in energy deficient states (such as AN and exercise-induced FHA) due to preferential differentiation of mesenchymal stem cells to adipocytes (at the expense of osteoblasts) and this increase correlates inversely with BMD (70) (71). *In vitro* studies demonstrate that bone marrow adipocytes release inflammatory cytokines and RANKL, which promote osteoclastogenesis, while the secretion of saturated fatty acids can also disrupt osteoblast function and lifespan (72–74). Putative mediators of the increase in BMAT include IGF-1, leptin, oestrogens, and pre-adipocyte factor-1 (75). Interestingly, a recent exploratory study in 16 women with FHA revealed that the expected increase in BMAT in this condition can be attenuated by transdermal 17β-estradiol treatment (71). Further studies in this respect and with control groups will be of great interest.

### Low Sodium

Lower circulating sodium levels are a frequent feature of AN (with or without amenorrhoea). In a large cross-sectional study of over 400 women with AN, a lower sodium level (<140mmol/L) was associated with a lower BMD at both the spine and hip compared to those with a sodium level >140mmol/L (reference range: 135-145mmol/L) (76). Overt hyponatraemia is also a recognised risk factor for bone loss, osteoporosis and fractures (77). Bone loss in hyponatraemia has been attributed to mobilisation of sodium stores from the bone *via* increased bone resorption (in an attempt to correct the low sodium), inappropriate vasopressin secretion and a direct effect of hyponatraemia on osteoclast activity (78).

In summary, patients with FHA have a multitude of endocrine abnormalities (beyond oestrogen deficiency) that can contribute to the disruption of skeletal homeostasis, as illustrated in **Figure 1**.

## Treatment

### Weight Gain, Restoration of Energy Balance, Reduction in Psychological Stress

Weight gain, restoration of energy balance and reduction in psychological stress leading to restoration of menstrual cycles are the most effective management strategies for FHA-related bone loss (79). In a study by Miller et al. involving 75 women with AN, weight gain especially lean body mass and resumption of menstrual function were both necessary for BMD recovery at the spine and hips (80). In contrast, improvement in BMD with weight restoration but without restoration of menses has been observed (14, 81), while others did not observe any change in BMD following weight gain alone (82, 83). These latter discrepant findings may be due to limited numbers, lack of controls, non-randomised study design and limited follow-up time, which may be insufficient to capture changes in BMD. However, it is worth noting that even if no incremental effect of weight gain was reported on BMD in some studies, a deterioration over time was nevertheless not observed, which is in itself a positive outcome (82, 83).

Unfortunately, achieving and maintaining a positive energy balance long-term is challenging for most women with FHA. Indeed, only about 60% of women with AN achieve recovery at 22 years (84). Additionally, AN is associated with a long-term increased risk of fractures in later life, irrespective of recovery (9). Even in athlete-related amenorrhoea, non-pharmacological intervention (increased dietary intake and/or decreased exercise) led to return of menses in only 17.6% of college athletes, while in the recent randomised controlled ‘REFUEL’ study, an increase in energy intake of about 330 kcal/day in exercising women with oligo/amenorrhea improved menstrual function in only 64% at 1 year (85, 86). Hence, there is a compelling need for effective long-term pharmacological replacement/treatment for women with FHA to protect their bones as the aforementioned non-pharmacological methods are challenging and not always fully effective.

### Oestrogen Treatment

Oestrogen replacement/treatment studies in FHA have revealed notable bone results related to the route, formulation and dosage of oestrogen. An up-to-date summary of clinical trials and other key studies related to bone treatment are reported in **Table 1**.

**Table 1:** Up-to-date summary of oestrogen treatment studies and other hormonal/pharmacological interventional studies in women with FHA.

	Study	Study Design	Subjects	Aetiology of FHA	Age(years)	Duration (months)	Intervention	Change in BMD
**Oestrogen Treatment**								
1	Hergenroeder et al, 1997 (87)	RCT	24	Mixed (AN/Athletes and Ballet dancers)	14-28	12	COCP (35μg EE + 0.5- 1.0mg norethindrone) vs 10mg medroxyprogeterone (MP) for 10 days vs placebo	Change in lumbar BMD: 5.4% (COCP) vs -10.2% (MP) vs -0.7% (placebo). This increase in BMD with COCP was significant compared to MP and placebo. Change in Femoral Neck BMD: 2.2% (COCP) vs -5.6% (MP) vs -2.7% (placebo). Not significantly different.
2	Gibson et al, 1999 (88)	RCT	34	Exercise (Runners)	18-34	12	Trisequens oral HRT (estriol 1mg + estradiol 2mg for 12 days, estriol 1mg + estradiol 2mg + norethisterone 1mg for 10 days, estriol 0.5mg + estradiol 1mg for 6 days) vs placebo	Change in lumbar BMD: 4.1% (in those who became eumenorrhoeic on HRT). Change in hip BMD: 3.8% (in those who became eumenorrheoic on HRT). Mean change in lumbar BMD relative to placebo: 1.5% (effect reflects return of menses in placebo + withdrawals from treatment group).
3	Castelo-Blanco et al, 2001 (89)	RCT	64	Not specified	Mean 24.4	12	COCP (30μg of EE + 0.15mg desogestrel) vs COCP (15μg of EE + 0.15mg desogestrel) vs placebo	Change in lumbar BMD: non-significant increase of 2.4% and 2.5% (COCP 30 μg and 15 μg cohorts respectively) vs -1.2% (placebo)
4	Grinspoon et al, 2002 (90)	RCT	66	AN	18-38	9	COCP (35μg EE + 0.4mg norethindrone) and recombinant human IGF-1 (rhIGF-1) or rhIGF-1 alone or COCP alone or placebo	Changes in lumbar BMD: 1.8% (COCP + rhIGF-1), vs 0.3% (rhIGF-1) vs -0.2% (COCP) vs -1.0% (placebo). Increase in BMD with COCP + rhIGF-1 was significantly higher relative to placebo only.
5	Warren et al, 2003 (91)	RCT	24	Exercise (ballet dancers)	Mean 20.8	24	Oral conjugated oestrogen (CE), Premarin (0.625mg) for 25 days with Provera 10mg for 10 days vs placebo and vs controls (ballet dancers with normal menses)	Changes in lumbar BMD: 5.6% (CE) vs 4.5% (placebo) vs 6.7% (controls). Not significantly different.
6	Rickenlund et al, 2004 (92)	Prospective-placebo controlled	26	Athletes (Endurance sports)	16-35	10	COCP (30μg EE + 150μg levonorgestrel) vs placebo	Small significant increase in total body BMD with COCP (but significant weight gain among subjects during study). No change in lumbar BMD with COCP
7	Warren et al, 2005 (93)	Open-labelled single arm extension study.	45	Not specified (but AN excluded)	18-40	10	COCP (35μg EE + 180-250 μg norgestimate)	Change in lumbar BMD: 1.5% (COCP). Significantly higher relative to baseline.
8	Strokosch et al., 2006 (94)	RCT	112	AN	11-17	13	COCP (35μg of EE + 0.18-0.28mg of norgestimate) or placebo	Change in lumbar BMD: 3.1% (COCP) vs 2.4% (placebo). Not significantly different. Change in hip BMD: 1.5% (COCP) vs 1.8% (placebo). Not significantly different.
9	Cobb et al, 2007 (95)	RCT	150	Exercise (runners)	18-26	24	COCP (35μg EE + 0.3mg norgestrel) or control (no intervention given)	Change in lumbar BMD: 1% per year (COCP, who remain amenorrhoeic). This increase in BMD was comparable to those who spontaneously regain menses but higher than those who did not in the control group.
10	Misra et al, 2011 (96)	RCT	110	AN	12-18	18	Transdermal 100mcg 17β-estradiol (TE) + medroxyprogesterone 2.5mg for 10 days vs placebo	Change in lumbar BMD: 2.6% (TE) vs 0.3% (placebo). This difference was significant. Change in BMD at hip: 0.004% (TE) vs -1.2% (placebo). This difference was significant.
11	Ackerman et al, 2019 (97)	RCT	121	Exercise	14-25	12	TE (100mcg 17β-estradiol + micronized progesterone 200mg) vs COCP (35μg EE + 0.15mg desogestrel) vs placebo	Change in lumbar BMD: 2.75% (TE) vs 0.3% (COCP, estimated from Graph) vs no change (placebo). Change in neck of hip BMD: 5.25% (TE) vs 1.8% (COCP, estimated from graph) vs 2% (placebo, estimated from graph).
12	Resulaj et al, 2020 (98)	Single-arm prospective	11	AN	Mean 37.2	6	TE (45mcg/day 17β-estradiol + levonorgestrel 0.015mg)	Significant increase in lumbar BMD by 2%.
**Androgen Treatment**								
1	Gordon et al, 2002 (99)	RCT	61	AN	14-28	12	DHEAS 50mg/day vs COCP 20μg EE + 0.1mg levonorgestrel vs placebo	Change in hip BMD: 1.7% (DHEAS and COCP). This was not significant when controlled for weight gain. No change in lumbar BMD.
2	Divasta et al, 2012 (100)	RCT	94	AN	13-27	18	DHEAS 50mg/day with COCP 20μg EE + 0.1mg levonorgestrel vs placebo	No change in lumbar or hip BMD in DHEAS with COCP or placebo groups.
3	Miller et al, 2011 (101)	RCT	77	AN	Mean: 25.3 (Risedronate), 27.1 (Testosterone), 25.2 (Combined), 26.9 (double-placebo)	12	Risedronate 35mg weekly vs testosterone 150μg daily patch vs risedronate 35mg weekly + testosterone 150μg daily vs placebo	No significant change in lumber and hip BMD with testosterone. Significant increase in lumbar BMD by 4% in risedronate group only, compared to placebo. Significant increase in hip BMD by 2% in risedronate group only, compared to placebo.
**Leptin Treatment**								
1	Welt et al, 2004 (102)	Prospective- placebo controlled	8	Exercise	19-33	3	r-metHuLeptin (0.08mg/kg) s.c daily vs placebo	No change in total body BMD.
2	Sienkiewicz, et al. (103)	RCT	20	Exercise	18-35	Up to 24	Metreleptin (0.08-0.12mg/kg/day) vs placebo	Significant increase in lumbar BMD by 4-6% from baseline. No change in hip BMD.
**Denosumab Treatment**								
1	Isobe et al	Retrospective case-series	3	AN	36-42	24	Denosumab 60 mg	Changes in lumbar BMD: 15.7% (case 1), 18.6% (case 2) and N/A (case 3) relative to baseline Changes in hip BMD: 36.1% (case 1), 11.6% (case 2), 10.7% (case 3) relative to baseline
**Bisphosphonate Treatment**								
1	Golden et al, 2005 (14)	RCT	32	AN	12-21	12	Alendronate 10mg daily vs placebo	Changes in lumbar spine: 3.5% (alendronate) vs 2.2% (placebo). Not significantly different. Change in Femoral neck BMD: 4.4% (alendronate) vs 2.3% (placebo). Not significantly different.
2	Miller et al, 2011 (101)	RCT	77	AN	Mean: 25.3 (risedronate), 27.1 (testosterone), 25.2 (combined), 26.9 (double-placebo)	12	Risedronate 35mg weekly vs testosterone 150 μg daily patch vs risedronate 35 mg weekly + Testosterone 150μg daily vs placebo	Significant increase in lumbar BMD by 4% in risedronate group only, compared to placebo. Significant increase in hip BMD by 2% in risedronate group only, compared to placebo. No significant change in lumbar and hip BMD with testosterone.
3	Miller et al, 2004 (104)	Prospective-placebo controlled	10	AN	Mean: 28.6 (risedronate), 26.9 (placebo)	9	5mg risedronate daily vs placebo	Significant increase in lumbar BMD by 4.9%, compared to placebo.
**Teriparatide Treatment**								
1	Fazeli et al, 2014 (105)	RCT	21	AN	Mean 47	6	Teriparatide (20μg SC daily) or placebo	Significant increase in lumbar BMD by 10.5% compared to placebo. No significant changes in BMD at hip.
2	Milos et al, 2020 (106)	Prospective single-arm	10	AN	21-33	24	Teriparatide (20μg SC daily)	Significant increase in lumbar BMD by 13.5%. Significant increase in femoral neck BMD by 5.0%.

RCT, Randomised Clinical Trial; EE, Ethinyl Estradiol; TE, Transdermal Oestrogen; vs, versus; SC, Subcutaneously; N/A, Not Available.

In a recent pivotal study, 121 oligo-amenorrhoeic athletes, aged 14-25 years, were randomised to a transdermal patch providing a ‘physiological’ 100 mcg 17β-estradiol, a combined oral contraceptive pill (COCP, containing a ‘supraphysiological’ 30 µg Ethinyl-Estradiol (EE)) or no oestrogen. Only the transdermal patch group exhibited BMD improvements at 12 months (approximately 3% at the lumbar spine and 5% at the femoral neck). Surprisingly, those on the COCP had a (nonsignificant) trend to a worse BMD compared to controls mainly at the total hip (97). Crucially, there were no significant differences in weight or menstrual function change between the patch and pill groups by the end of the study, that could have confounded these results. Microarchitectural indices also improved significantly in the patch versus COCP group, especially at the tibia (107). These aforementioned findings in oligo-amenorrhoeic athletes are mirrored in females with AN. Misra et al. showed that 18 months of transdermal 17β-estradiol (100 mcg patch twice weekly) but not the COCP (35 µg of EE + 0.18-0.28 mg of norgestimate) led to an improvement of 2.6% in lumbar BMD in adolescents with AN (96). In a separate group of adolescents with AN, treatment with a triphasic COCP (35 µg of EE + 0.18–0.25 mg of norgestimate) for 13 months, did not lead to any significant change in lumbar or hip BMD (94). Similarly, in a recent 6-month pilot study, Resulaj et al. observed an increase of 2% in the lumbar BMD of women with AN (mean age 37 years), following transdermal oestradiol (45 mcg/day), although there was no control group (98). In contrast to transdermal physiological dose oestrogen, the COCP has not shown any convincing benefits (in terms of BMD) in adult women with FHA due to AN or exercise (90, 95).

These differing actions of oestrogen treatment have been mainly attributed to the route of its administration. Oral COCP inhibits IGF-1 production *via* first-pass hepatic metabolism, from which transdermal oestrogen is exempt. Indeed, a reduction in IGF-1 levels, associated with a greater fall in P1NP (a marker of osteoblastic activity) levels is observed during COCP treatment, but not with transdermal 17β-estradiol. Although the oestrogen dose is higher in studies of the COCP compared to transdermal oestrogen, even lower oral doses of oestrogen (1mg 17β-oestradiol) have suppressive effects on IGF-1 compared to transdermal oestrogen (108). Furthermore, oral oestrogens can increase hepatic sex hormone binding globulin levels, thereby reducing bioavailable oestrogen to the detriment of skeletal homeostasis (109).

In summary, the body of evidence for the positive effect of oestrogen treatment on bone in FHA defines a beneficial effect for transdermal oestrogen replacement over the COCP, with promising recent results (96, 97). This concept was confirmed in a very recent meta-analysis of the effects of oral contraceptives, conjugated oestrogens and transdermal oestrogens in FHA, with the latter showing consistent superiority in terms of BMD gains (110). However, it is worth noting inherent difficulties in these studies, with small numbers, high drop-out rates, relatively short follow-up, heterogeneity in types and doses of oestrogen (and progestin) used, and crucially the lack of fracture-related data. Therefore, further work is warranted to assess the doses (physiological (i.e. replacement) versus supraphysiological), the types (17β-estradiol versus ethinyl-oestradiol versus conjugated oestrogens) and the routes of administration of oestrogen (transdermal versus oral) to clearly define the optimal treatment strategy. Currently, the data point to transdermal oestrogen replacement as the optimal strategy.

### Androgen Treatment

Transdermal testosterone replacement and DHEA do not increase BMD in women with AN (with and without amenorrhoea) at 12 months (**Table 1**) (99, 101). However, a combination of DHEA and COCP led to stabilisation of BMD over 18 months, relative to placebo, where a drop in BMD was observed (100). Further studies are required to clarify the independent benefits of androgen treatment.

### IGF1 Treatment

Given the aforementioned suppression of IGF1 observed in FHA, it is not surprising that recombinant human IGF-1 (in combination with a COCP), led to an increase in lumbar BMD compared to placebo, by 1.8% versus -1% respectively at 9 months in women with AN and osteoporosis (aged between 18-38 years). The corresponding changes in lumbar BMD with IGF-1 or COCP monotherapy were 0.3% and -0.2% respectively (See **Table 1**). Longer studies of IGF-1 treatment are warranted given that the duration was only 9 months (90).

### Leptin Treatment

Leptin treatment has also been the subject of study in FHA. Subcutaneous leptin administration can restore reproductive axis function with return of menses in a third of women with FHA (due to AN) with associated reductions in cortisol, and increases in IGF-1, thyroid hormones and bone formation markers (102). A 2-year study with daily subcutaneous metreleptin injection, culminated in 4-6% gain in BMD at the lumbar spine in exercising women (103). However, leptin treatment was associated with approximately 3% weight loss which has ultimately restricted its development for FHA despite these promising biochemical and bone outcomes. See **Table 1** for a summary of studies investigating leptin treatment in FHA.

### Bisphosphonates and Denosumab

There is a limited number of studies evaluating the benefits of bisphosphonates in FHA-related bone loss. These are small prospective studies looking at alendronate, risedronate and etidonate (14, 101, 111). Only risedronate showed a significant increase in BMD at the spine and hip by approximately 4% and 2% respectively, at 9-12 months in women with AN, most of whom were not experiencing endogenous menses (101, 104). However, no positive effect of bisphosphonates has been observed in adolescents with AN (14); presumably due to reduced underlying bone turnover as discussed previously. Key points of these studies are outlined in **Table 1**.

There are case reports supporting the use of denosumab in osteoporotic women with AN (aged 37-42 years, BMI 12.2-18.3 kg/m^2^) although menstrual status was not reported (112). However, no clinical trials have investigated denosumab in FHA to-date.

The barriers to using bisphosphonates in FHA are their prolonged half-lives with a small but potential teratogenic (observed in rodent studies but not consistently in humans) or neonatal complication risk, in a patient population often in their reproductive years (113). Similarly, denosumab is associated with complications if used in pregnancy (114).

### Teriparatide

Anabolic agents such as teriparatide have been trialled with good effect (See **Table 1**). In a randomised controlled trial (RCT) of 21 osteoporotic women with AN and a mean BMI of 17.6 kg/m^2^, teriparatide resulted in a significant increase in lumbar BMD of 6% at only 6 months (105). More recently, Milos et al. provided further supporting evidence by studying a slightly younger cohort of women with AN (mean BMI 15.6 kg/m^2^) with or without previous fragility fractures. Teriparatide treatment for 24 months resulted in a significant increase in BMD of 13.5% at the lumbar spine and 5% at the hip. Notably, this was independent of gain in body weight and body fat (106). However, it is worth noting that this study lacked a control group and changes in menstrual function, which may have confounded the results, were not reported. Barriers to the use of teriparatide are its limited use of up to 2 years (which may lead the clinician to reserve teriparatide for when the patient with FHA is older or use it for shorter periods at different ages), cost and the inconvenience of daily injections.

## Future Avenues

### Romosozumab

Future pharmaceutical avenues include the humanised monoclonal antibody to sclerostin, Romosozumab, which is approved for the treatment of post-menopausal osteoporosis. Of note sclerostin levels have been reported as unaltered or raised in adolescent and young women with AN compared to healthy controls (115, 116). Data in FHA are awaited but this suggests that women with FHA (at least due to AN) may be susceptible to sclerostin pathway inhibition. Studies are therefore warranted in this regard although safety in women of reproductive age will again need to be clearly ascertained (there are no human pregnancy data as yet).

### Kisspeptin

Another recent promising avenue is kisspeptin treatment. It has previously been demonstrated that kisspeptin administration can restore LH pulsatility in women with FHA acutely while twice weekly injections for 8 weeks can stimulate the secretion of reproductive hormones without significant desensitisation (117, 118). Recent data has now emerged from a bone perspective suggesting that kisspeptin administration also can have direct positive effects in human bones. In this study we showed that kisspeptin potently stimulated osteogenic differentiation of osteoblast progenitors and inhibited bone resorption *in vitro* (by up to 53.4%), in a dose-dependent manner. Furthermore, acute kisspeptin administration to healthy young men increased osteoblast activity *in vivo*. Further studies are warranted but collectively these data suggest that kisspeptin administration could benefit skeletal homeostasis in FHA by restoring reproductive hormone secretion as well as by direct effects on bone.

### Gut Microbiota

Another emerging avenue is the association of the gut microbiota with abnormal body weight. Signature changes recently reported in women with AN include a relative reduction in firmicutes and short-chain fatty acids (SCFA), and an increase in bacteroidetes, Methanobrevibacter smithii and Escherichia coli (E.coli) species (119). Some of these changes may be adaptive but a positive association between E.coli and appetite suppression at the level of the MC4 receptors has been described in rodents (120). Yan et al. demonstrated that treatment with broad spectrum antibiotic for 2 months led to depletion of the microbiota in female germ-free mice with subsequent reduction in SCFA and IGF-1 levels. In contrast, SCFA supplementation in antibiotic-treated mice for 6 weeks, restored levels of IGF-1 and improved bone mass to reflect that of non-antibiotic-treated mice (121). Further clinical studies, specifically exploring the role of SCFA and pro and pre-biotics as potential treatment agents for bone health in FHA are now warranted.

## Conclusion

Low BMD with an increased risk of fractures is a major complication of FHA due to a multitude of factors as updated above. Given the undoubted severity of the negative effects on bones, there remains an unmet need to clearly determine the optimal oestrogen replacement strategy as well as testing alternative and new pharmacological interventions to treat FHA-related bone loss. Current evidence favours transdermal 17β-estradiol as being the most promising intervention from an oestrogen replacement perspective, although larger and longer studies are needed to verify its long-term benefits, especially on the ultimate outcome of fractures. In addition, the potential use of romosozumab, kisspeptin and pro/prebiotics, warrant further exploration.

## Author Contributions

PB drafted the manuscript. AC reviewed and amended the manuscript. All authors contributed to the article and approved the submitted version.

## Funding

The Endocrine Bone Unit is funded by the National Health Service (NHS). The Section of Endocrinology and Investigative Medicine is funded by grants from the MRC, NIHR and is supported by the NIHR Biomedical Research Centre Funding Scheme and the NIHR/Imperial Clinical Research Facility. The views expressed are those of the authors and not necessarily those of the NHS, the NIHR or the Department of Health. PB and AC are supported by the NHS.

## Conflict of Interest

AC has received non-promotional educational lecture honoraria and conference support from Amgen.

The remaining author declares that the research was conducted in the absence of any commercial or financial relationships that could be construed as a potential conflict of interest.

## Publisher’s Note

All claims expressed in this article are solely those of the authors and do not necessarily represent those of their affiliated organizations, or those of the publisher, the editors and the reviewers. Any product that may be evaluated in this article, or claim that may be made by its manufacturer, is not guaranteed or endorsed by the publisher.
